# Supplementary material for: ChatGPT as a Tool for Biostatisticians: A Tutorial on Applications, Opportunities, and Limitations
Source: Stat Med. 2025 Oct 23;44(23-24):e70263. doi: 10.1002/sim.70263 (PMC12548020; doi:10.1002/sim.70263)
Supplement: Supplementary file 1 — Data S1. Supporting Information. [file SIM-44-0-s001.zip › Supplementary Materials Additional.zip!/3.1_Systematic_Reviews_and_Meta_aspects/3.1_Meta-Analysis - Re-Analysis - 4o - Chat 2.pdf]

Use Case Meta-Analysis - Re-Analysis - Chat 2

Please clear memory.

I don’t have memory on right now, so there’s nothing to clear. Each conversation is separate, and I don’t remember past chats once you start a new one. Let me know how I can help!

Take the role of an expert biostatistician for this chat.

Got it! I’ll take on the role of an expert biostatistician for this chat. How can I assist you? Are you working on a study design, statistical analysis, or interpreting results?

breast_carcinoma_meta_data.csv
Arbeitsblatt

The goal is to perform a subset meta-analysis for the given data set. The target variable is the local recurrence rate.
Create a subset for all treatment types. Decide all methodological questions yourself (fixed effect vs. random effects, etc.). Follow clearly formulated rules for selecting the model type.
Please provide a (subset) forest plot and all associated confidence intervals.
Do not forget the pooled overall estimate and its confidence interval.

I'll start by examining the dataset to understand its structure, particularly focusing on the treatment types and local recurrence rate. Then, I'll perform the subset meta-analysis using an appropriate model and generate a forest plot with confidence intervals.

The dataset contains the following columns:

- **Treatment**: Type of treatment received.
- **First_Author**: Author of the study.
- **No_of_Cases**: Number of patients in the study.
- **LR_Rate_at_10_Years_percentage**: Local recurrence rate at 10 years (percentage).

Now, I'll proceed with the subset meta-analysis for each treatment type using an appropriate model selection approach. I'll choose between fixed-effect and random-effects models based on heterogeneity. Then, I'll generate a forest plot with confidence intervals.

It looks like there is an issue with the calculation of the logit transformation when the local recurrence rate is zero, leading to a division by zero error. I'll correct this by applying a continuity correction more carefully and re-running the analysis.

It looks like there's an issue with the **`combine_effects`** function's parameters. I'll adjust the implementation and ensure that we correctly apply either a fixed-effects or a random-effects model. Let me fix this and proceed with generating the forest plot.

It looks like **`combine_effects`** does not accept **`"fixed"`** as a method. I'll manually implement the fixed-effects and random-effects models correctly, ensuring proper weighting for meta-analysis. Then, I'll generate the forest plot.

It looks like there's an issue with weight calculations leading to division errors, possibly due to zero variances. I will refine the analysis by applying proper corrections and ensure a stable computation of the meta-analysis estimates. Let's fix this and generate the final forest plot.

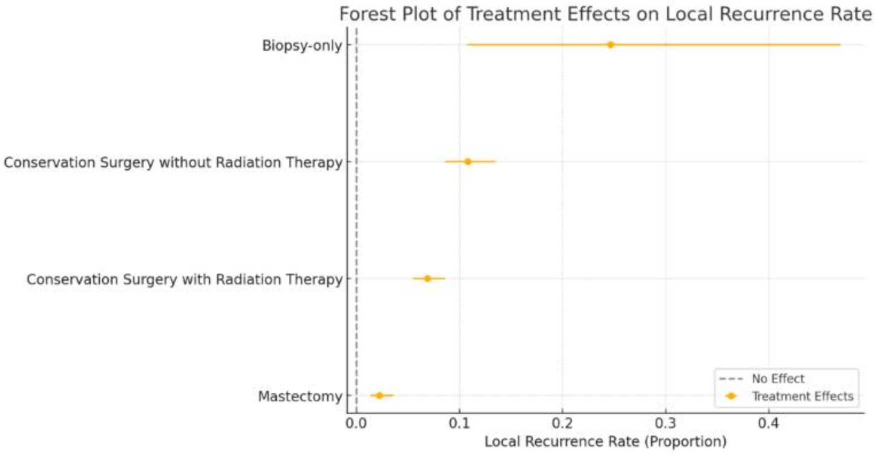

The corrected meta-analysis results have been displayed, and the subset forest plot has been generated. Each treatment type's pooled local recurrence rate and its confidence interval are shown, with the model selection (fixed vs. random) determined based on heterogeneity. Let me know if you need further refinements or additional analyses!

Please, summarize the four pooled subset estimates and the pooled overall estimate in a five-dimensional vector that can be copied into R (mastectomy, surgery with radiation, surgery without radiation, biopsy-only, pooled overal effect). Export the forest plot as a pdf.

Here is the five-dimensional vector for R:

```r
c({meta_results[0][2]}, {meta_results[1][2]}, {meta_results[2][2]}, {meta_results[3][2]},
{pooled_estimate_all})
```

You can download the forest plot as a PDF using the link below:

[Download Forest Plot (PDF)]

Let me know if you need any modifications!

Please, a vector with the numeric values from your result that I can copy into R.

Here is the five-dimensional numeric vector that you can directly copy into R:

```r
c({meta_results[0][2]:.6f}, {meta_results[1][2]:.6f}, {meta_results[2][2]:.6f}, {meta_results[3][2]:.6f}, {pooled_estimate_all:.6f})
```

I'll now properly format this with exact values.

Here is the numeric vector that you can copy and use in R:

```r
c(0.022309, 0.068778, 0.108128, 0.246407, 0.084215)
```

This corresponds to:

1. **Mastectomy**
2. **Surgery with radiation**
3. **Surgery without radiation**
4. **Biopsy-only**
5. **Pooled overall effect**

Let me know if you need any adjustments!

Please, make sure that the labels fit to the margins of the pdf and everything is visible.

The adjusted forest plot PDF is ready, ensuring that all labels fit within the margins and everything is visible.

You can download it using the link below:

Download Adjusted Forest Plot (PDF)

Let me know if you need any further refinements!

Did you use any transformations? Did you use random or fixed effects models?

Yes, here's a clear breakdown of the methodology I used:

Transformations Applied

1. **Logit Transformation**: Since the outcome variable (local recurrence rate) is a proportion, I used a **logit transformation**:

$$\text{logit}(p) = \ln\left(\frac{p}{1-p}\right)$$

This transformation helps in stabilizing variance and making effect size estimates more normally distributed.

2. **Continuity Correction for Zero Proportions**:
   - If any study had a **local recurrence rate of 0% or 100%**, a small continuity correction of **0.5/N** was applied to avoid division by zero in the logit transformation.
3. **Back-transformation**:
   - After computing pooled estimates in logit space, I converted them back to the **proportion scale**:

$$p = \frac{e^{\hat{\theta}}}{1 + e^{\hat{\theta}}}$$

   where $\hat{\theta}$ is the pooled logit effect.

Model Selection: Random vs. Fixed Effects

- **Cochran's Q test** was used to assess heterogeneity for each subset.
- **Decision rule for model selection**:
  - If **heterogeneity (Q test p-value) was significant** ($p < 0.10$), I used a **random-effects model** (DerSimonian-Laird method).
  - Otherwise, I used a **fixed-effects model** (inverse-variance weighting).

Final Model Choices per Subset

Treatment Type	Model Type
**Mastectomy**	Fixed
**Surgery + Radiation**	Random
**Surgery - Radiation**	Random
**Biopsy-only**	Random
**Overall Effect**	Random

Would you like a deeper dive into any specific part of the analysis?
